# rs1859168 A > C polymorphism regulates HOTTIP expression and reduces risk of pancreatic cancer in a Chinese population

**DOI:** 10.1186/s12957-017-1218-0

**Published:** 2017-08-17

**Authors:** Pinghai Hu, Ou Qiao, Jun Wang, Jiao Li, Hao Jin, Zhaolian Li, Yan Jin

**Affiliations:** Department of Hepatobiliary Surgery, First People’s Hospital of Yunnan Province, No. 157 Jinbi Rd., Xishan District, Kunming, 6050032 China

**Keywords:** lncRNA, Hottip, Polymorphism, Pancreatic cancer

## Abstract

**Background:**

Long non-coding RNAs (lncRNAs) are aberrantly expressed in many types of human cancer including pancreatic cancer (PC) and correlated with tumorigenesis and cancer prognosis, whereas knowledge about regulatory mechanism of lncRNA expression is few known. This study aimed to explore whether polymorphisms in lncRNAs genes are associated with PC susceptibility by affecting its expression.

**Methods:**

We first genotyped three common single-nucleotide polymorphisms (SNPs) of lncRNA genes (HOTTIP rs1859168, HOTAIR rs4759314, and H19 rs217727) in 416 paired PC patients and controls, and then validated the results in another 505 paired PC patients and controls. The genotype-phenotype correlation was examined in 50 PC tissue samples with different genotypes as well as by luciferase reporter assay.

**Results:**

In the discovery set, only the HOTTIP rs1859168 A > C showed to be significantly associated with a reduced PC risk (CC vs AA: odds ratio (OR) = 0.71, 95% confidence interval (95%CI) = 0.57–0.88, *P* = 0.002; recessive model: adjusted OR = 0.51, 95%CI = 0.38–0.68, *P* < 0.001; additive model: adjusted OR = 0.67, 95%CI = 0.51–0.82, *P* < 0.001). The results in validation set and pooled population also indicated that the C allele of HOTTIP rs1859168 could significantly decrease the risk of PC. In addition, the genotype-phenotype association analysis suggested that HOTTIP expression level was significantly lower in PC samples with CC genotype than that in samples with AA and AC genotype. Furthermore, the C allele of HOTTIP rs1859168 could significantly decrease the relative luciferase activity compared to the A allele in three PC cell lines.

**Conclusions:**

The current findings provided evidence that the functional rs1859168 A > C polymorphism may decrease the PC risk by down-regulating the HOTTIP expression.

## Background

Pancreatic cancer (PC) is the sixth leading causes of cancer-related deaths in China [1]. According to 2015 Cancer Statistics, the incidence rate of PC was 90.1 per 100,000 and the mortality rate was 79.4 per 100,000 [1]. Moreover, the prognosis for PC patients is ultimately poor due to delay in diagnosis, rapid progress, and conventional treatment resistance [2–4]. Thus, PC had been considered as one of the most serious health threats in China. PC is a complex disease caused by a variety of genetic and environmental factors. Previous epidemiological studies have identified several risk factors for PC morbidity, including high fat and protein diets, smoking, chronic pancreatitis, diabetes mellitus, and family history of cancer [5]. Recently, numerous reports have shown that genomics and epigenetics play risk-modulating roles in the susceptibility, progression, and prognosis of PC.

Long non-coding RNAs (lncRNAs), whose length are more than 200 nucleotides and cannot code protein [6, 7], are getting more and more attention as the important role in the transcriptional, epigenetic, and post-transcriptional regulation of gene expression [7, 8]. A lot of studies have suggested that lncRNAs could deliver function in varieties of cellular processes, including cell cycle regulation, cell differentiation, proliferation, growth, and apoptosis [9–11]. In addition, increasing numbers of studies revealed that the abnormal lncRNA level is closely related to the susceptibility, development, progression, and prognosis of various human cancers including PC [12–15]. Previous studies have showed that three key lncRNAs (HOTTIP, HOTAIR, and H19) might serve as candidate genes for cancerization and therapeutic targets [12, 16–21].

A growing number of evidence has demonstrated that it plays a vital role in the process of carcinogenesis that the expression and function of several key lncRNAs are affected by single-nucleotide polymorphisms (SNPs) [22–24]. To date, a large number of studies have explored the association between the SNPs in lncRNAs and cancer susceptibility. For instance, functional SNP rs4759314 in HOTAIR gene could affect the expression of HOTAIR, and upregulation of HOTAIR was significantly associated with development, progression, and prognosis of various cancers [25–27]. Xia et al. demonstrated the significant association between rs217727 polymorphism and breast cancer risk from 464 breast cancer patients and 467 controls [28]. In addition, functional prediction of SNP revealed that rs1859168 could alter HOTTIP expression by influencing the transcription factor binding sites, and HOTTIP takes part in regulating cancer cell proliferation and survival [29–31]. Furthermore, several meta-analyses about the associations between SNPs in these key lncRNAs genes and various cancers risk have been conducted [32, 33]. However, the results were inconsistent and there were no studies focused on the problem which is the associations between these SNPs and PC risk.

Considering the vital roles of lncRNAs for cancer development, we enforced a two-stage case-control study to evaluate the associations of three common SNPs (HOTTIP rs1859168 A > C, HOTAIR rs4759314 A > G, and H19 rs217727 C > T) in key lncRNAs genes with PC risk in China, and explore the biological mechanism of the SNPs.

## Methods

### Sample selection and design

This study was approved by the Ethics Committee of the First People’s Hospital of Yunnan Province (Kunming, China) and written informed consent was also obtained from each participant after a clear explanation about study objective. The study was composed of two independent case-control populations, the discovery set and the validation set, including a total of 921 PC cases and 921 age and gender matched healthy controls. All subjects were genetically unrelated to Han Chinese people. The 416 PC patients were diagnosed and treated at the Department of Hepatobiliary Surgery, First People’s Hospital of Yunnan Province (Kunming, China) from 2010 to 2015; the other 505 PC patients were enrolled from the Department of Hepatobiliary Surgery, First Affiliated Hospital of Kunming Medical University (Kunming, China) from 2011 to 2015. All of PC patients in both populations were consecutively recruited. All patients were newly diagnosed, pathologically confirmed and without treatment of any medications. The diagnosis of PC was confirmed by histopathological or cytological analysis according to the World Health Organization classification. Another total of 921 subjects (416 from discover set and 505 from validation set) without cancer or other serious illness were selected as controls, and they were matched with the PC patients by age (± 5 years), sex, and area of residence (urban and rural).

Information on demographic characteristics and risk factors of participants were collected using structured questionnaires, including smoking status, alcohol intake, pancreatitis, history of hypertension and diabetes mellitus, family history of cancer. Fasting peripheral venous blood samples from patients and controls were collected in sterile tubes with EDTA-Na_2_ anticoagulant. All blood samples were stored at −80 °C until use. In addition, before receiving any treatment, 50 patients underwent surgery to remove PC tumor tissue and these fresh tissue samples were stored in liquid nitrogen.

### Genotyping of lncRNA genes

Genomic DNA was extracted from 200-ul EDTA-Na_2_ anticoagulant venous blood sample from each participant using genomic DNA purification kit (Tiangen, China) as described in the manufacturer’s instructions. According to the manufacturer’s instructions (Applied Biosystems), TaqMan SNP Genotyping Assay and ABI 7900HT Real-Time PCR System were used to genotype three polymorphisms (HOTTIP rs1859168 A > C, HOTAIR rs4759314 A > G, and H19 rs217727 C > T) in 921 PC patients and 921 controls. The primer sequences for each SNP were obtained from Applied Biosystems and described in Table 1. The quality control was done as follows: 3 duplicates including three negative control samples (water) were performed in each 96 well assay plate; genotypes analysis was performed in a blind fashion; 10% of the samples were randomly selected for repeated genotyping for confirmation, and the results were 100% concordant.

**Table 1:** General characteristics of genetic variants in long non-coding RNA genes

Gene	SNP	Chr: position	MAF	HWE	Primers
HOTTIP	rs1859168	Chr7: 27,202,740	A = 0.131	0.099	Sense: ACGTTGGATGAATGATAGGGACACATCGGG
					Antisense: ACGTTGGATGAGGTTTGTCTGAGAGGGATG
HOTAIR	rs4759314	Chr12: 53,968,051	G = 0.095	0.166	Sense: ATGATCTGCTTGGAAGGGATATAAA
					Antisense: GCCTGGGCTTTTTCAGGTTT
H19	rs217727	Chr11: 1,995,678	*T* = 0.320	0.302	Sense: ACTCAGGAATCGGCTCTGGAAGGTG
					Antisense: GATGTGGTGGCTGGTGGTCAACGGT

*MAF* minor allele frequency, *HWE* Hardy-Weinberg equilibrium

### Real-time PCR analysis

Total RNA was extracted from PC tissue samples using Trizol reagent (Invitrogen, USA) according to instruction. A Roche Light-Cycler (Roche, Basel, Switzerland) and SYBR Green reaction mix (Qiagen, Germany) were used to quantify relative HOTTIP expression, and GAPDH mRNA was chosen as a normalizing control. The primers for HOTTIP or GAPDH mRNA were as follows: HOTTIP forward: 5′-GTGGGGCCCAGACCCGC-3′; HOTTIP reverse: 5′-AATGATAGGGACACATCGGGGAACT-3′; GAPDH mRNA forward: 5′-ACCACAGTCCATGCCATCAC-3′; GAPDH mRNA reverse: 5′-TCCACCACCCTGTTGCTGTA-3′. The relative expression of HOTTIP was evaluated using the delta-delta CT (2^−ΔΔCt^) method.

### Luciferase reporter assay

As rs1859168 was associated with PC risk and HOTTIP expression, we evaluated whether this variant had allele-specific effect on its promoter activity. Luciferase reporter gene plasmid was constructed using the psiCHECK™-2 Vector (Promega). Firstly, we amplified the HOTTIP sequence with rs1859168 AA genotype by Genewiz Company (Suzhou, China) and cloned into pGL3-basic plasmids with Hind III and Bgl II digestion sites (Promega) yielding the wild-type vector. The mutant-type vector including the C allele of rs1859168 was generated using sit-specific mutagenesis (Stratagene). The resulting constructs were verified by direct sequencing. Then, the PC cell lines Panc-1, BxPC-3, and SW1990 was transfected with the constructed reporter plasmid and was cultured in Dulbecco’s modified Eagle’s medium using Attractene Transfection Reagent (QIAGEN). Finally, the cells were harvested after 24 h transfection and were determined for two luciferase activity (Renilla and Firefly) with the Dual-Luciferase Reporter Assay System (Promega).

### Statistical analysis

All data was analyzed using SPSS 21.0 software (Statistical Package for the Social Sciences, Chicago, USA) and when *P* < 0.05 was considered as significant (two-tailed). Firstly, the Pearson chi-square test and goodness-of-fit chi-square test were used for assessing differences in characteristics between two groups and for detecting Hardy-Weinberg equilibrium among the controls and patients for SNPs, respectively. Secondly, the multiple logistic regression analysis was performed to assess the relationship of each SNP and the risk of PC, with adjustment of age, gender, smoking status, alcohol intake, body mass index, hypertension, and history of pancreatitis, diabetes mellitus, and cancer. Lastly, comparing the relative expression levels of HOTTIP in PC tissues among different genotypic groups and luciferase activities in PC cell lines among different rs1859168 allele groups were carried out using Non-parametric Mann-Whitney *U* test and Student’s *t* test, respectively.

## Results

### Characteristics of the participants

A total of 921 PC patients and 921 unrelated gender and age-matched healthy controls were recruited in our study, including the discovery set (416 patients and 416 controls) and validation set (505 patients and 505 controls). The demographic characteristics for the PC patients and controls of both discovery and validation sets were showed in Table 2. The distributions of age, gender, body mass index, smoking status, alcohol intake, and hypertension status were not different between the PC patients and controls in both discovery and validation sets (*P* > 0.05). However, there were more individuals with history of pancreatitis and diabetes mellitus and family history of cancer in the PC patients than in the controls for both sets (*P* < 0.05).

**Table 2:** Characteristics of the study subjects

Variable	Discovery set			Validation set		
	patients	Controls	*P*	Patients	Controls	*P*
	(*N* = 416)	(*N* = 416)		(*N* = 505)	(*N* = 505)	
Age			0.532			0.184
< 60	191 (45.9)	200 (48.1)		217 (43.0)	238 (47.1)	
≥ 60	225 (54.1)	216 (51.9)		288 (57.0)	267 (52.9)	
Gender			0.357			0.477
Male	245 (58.9)	258 (62.0)		316 (62.6)	305 (60.4)	
Female	171 (41.1)	158 (38.0)		189 (37.4)	200 (39.6)	
Body mass index			0.329			0.613
< 23	178 (42.8)	192 (46.2)		227 (45.0)	235 (46.5)	
≥ 23	238 (57.2)	224 (53.8)		278 (55.0)	270 (53.5)	
Smoking status			0.363			0.334
No	229 (55.0)	242 (58.2)		298 (59.0)	313 (62.0)	
Yes	187 (45.0)	174 (41.8)		207 (41.0)	192 (38.0)	
Alcohol consumer			0.360			0.339
No	237 (57.0)	250 (60.1)		300 (59.4)	285 (56.4)	
Yes	179 (43.0)	166 (39.9)		205 (40.6)	220 (43.6)	
Hypertension			0.082			0.067
No	297 (71.4)	319 (76.7)		353 (69.9)	379 (75.0)	
Yes	119 (28.6)	97 (23.3)		152 (30.1)	126 (25.0)	
History of pancreatitis			0.003			0.001
No	400 (96.2)	413 (99.3)		481 (95.2)	499 (98.8)	
Yes	16 (3.8)	3 (0.7)		24 (4.8)	6 (1.2)	
History of diabetes mellitus			< 0.001			<0.001
No	332 (79.8)	383 (92.1)		409 (81.0)	460 (91.1)	
Yes	84 (20.2)	33 (7.9)		96 (19.0)	45 (8.9)	
Family history of cancer			0.002			0.003
No	394 (94.7)	410 (98.6)		480 (95.0)	497 (98.4)	
Yes	22 (5.3)	6 (1.4)		25 (5.0)	8 (1.6)	

### Associations between SNPs in lncRNA genes and PC risk

The observed genotypic frequencies of HOTTIP rs1859168, HOTAIR rs4759314, and H19 rs217727 in controls are all consistent with the Hardy-Weinberg equilibrium (*P* > 0.05). The genotype distributions of the three polymorphisms between PC patients and controls in the discovery population were shown in Table 3. Compared with HOTTIP rs1859168 AA genotype, subjects with the CC genotype obviously cut down the risk of PC (adjusted OR = 0.71, 95%CI = 0.57–0.88, *P* = 0.002). A significant association was found between rs1859168 and PC risk in recessive model (adjusted OR = 0.51, 95%CI = 0.38–0.68, *P* < 0.001) and additive model (adjusted OR = 0.67, 95%CI = 0.51–0.82, *P* < 0.001). However, HOTAIR rs4759314 A>G and H19 rs217727 C>T showed no significant associations with the risk of PC. HOTTIP rs1859168 was further replicated in validation set and was found to be significantly associated with decreased risk of PC (CC vs AA: adjusted OR = 0.70, 95%CI = 0.58–0.85, *P* < 0.001; dominant model: adjusted OR = 0.71, 95%CI = 0.51–0.99, *P* = 0.042; recessive model: OR = 0.56, 95%CI = 0.43–0.75, *P* < 0.001; additive model: OR = 0.69, 95%CI = 0.57–0.84, *P* < 0.001). When the two populations are pooled together, the results also indicated that the C allele of rs1859168 could meaningfully decrease PC incidence (CC vs AA: adjusted OR = 0.71, 95%CI = 0.61–0.81, *P* < 0.001; dominant model: adjusted OR = 0.72, 95%CI = 0.56–0.93, *P* = 0.010; recessive model: OR = 0.54, 95%CI = 0.44–0.66, *P* < 0.001; additive model: OR = 0.68, 95%CI = 0.59–0.78, *P* < 0.001)

**Table 3:** Distribution of long non-coding RNA genes in case and control subjects and their associations with risk of pancreatic cancer

Gene	SNPs	Patients	Controls	OR^a^ (95%CI)	*P*
		*N* (%)	*N* (%)		
Discovery set		*N* = 416	*N* = 416		
HOTTIP	rs1859168				
	AA	80 (19.2)	60 (14.4)	1	
	AC	220 (52.9)	175 (42.1)	0.99 (0.66–1.49)	0.973
	CC	116 (27.9)	181 (43.5)	0.71 (0.57–0.88)	0.002
	Dominant model			0.74 (0.51–1.08)	0.122
	Recessive model			0.51 (0.38–0.68)	< 0.001
	Additive model			0.67 (0.51–0.82)	< 0.001
HOTAIR	rs4759314				
	AA	333 (80.0)	325 (78.1)	1	
	AG	75 (18.0)	82 (19.7)	0.91 (0.64–1.31)	0.621
	GG	8 (2.0)	9 (2.2)	0.99 (0.60–1.64)	0.957
	Dominant model			0.92 (0.65–1.30)	0.621
	Recessive model			0.96 (0.35–2.61)	0.928
	Additive model			0.93 (0.69–1.26)	0.646
H19	rs217727				
	CC	133 (32.0)	128 (30.8)	1	
	CT	200 (48.0)	196 (47.1)	1.04 (0.75–1.44)	0.821
	TT	83 (20.0)	92 (22.1)	0.93 (0.76–1.13)	0.462
	Dominant model			0.98 (0.72–1.32)	0.877
	Recessive model			0.84 (0.59–1.19)	0.325
	Additive model			0.94 (0.78–1.14)	0.509
Validation set		*N* = 505	*N* = 505		
HOTTIP	rs1859168				
	AA	105 (20.8)	76 (15.0)	1	
	AC	277 (54.9)	246 (48.7)	0.86 (0.60–1.22)	0.387
	CC	123 (24.4)	183 (36.2)	0.70 (0.58–0.85)	< 0.001
	Dominant model			0.71 (0.51–0.99)	0.042
	Recessive model			0.56 (0.43–0.75)	< 0.001
	Additive model			0.69 (0.57–0.84)	< 0.001
Pooled analysis		*N* = 921	*N* = 921		
HOTTIP	rs1859168				
	AA	185 (20.1)	136 (14.8)	1	
	AC	497 (54.0)	421 (45.7)	0.92 (0.70–1.20)	0.522
	CC	239 (26.0)	364 (39.5)	0.71 (0.61–0.81)	< 0.001
	Dominant model			0.72 (0.56–0.93)	0.010
	Recessive model			0.54 (0.44–0.66)	< 0.001
	Additive model			0.68 (0.59–0.78)	< 0.001

ORs and *P* values were obtained from logistic regression models with adjustment for age, gender, body mass index, smoking status, alcohol intake, hypertension, history of pancreatitis, diabetes mellitus, and family history of cancer

### Stratification analysis for associations between HOTTIP rs1859168 and PC risk

The association between HOTTIP rs1859168 polymorphism and PC risk in the pooled populations was further evaluated by the stratified analysis. The associations between rs1859168 AC + CC genotype and decreased risk of PC were more obvious in populations with age longer than 60, males, non-smokers, drinkers, and populations with hypertension, no history of pancreatitis, no history of diabetes mellitus, and no family history of cancer than in their counterparts (Table 4).

**Table 4:** Stratification analysis of the association between rs1859168 genotypes and pancreatic cancer risk

Variables	rs1859168 (AA/AC + CC)			
	Patients	Control	OR (95%CI)	*P*
Age				
< 60	81/327	68/370	0.78 (0.54–1.13)	0.184
≥ 60	104/409	68/415	0.67 (0.48–0.95)	0.025
Sex				
Male	118/443	82/481	0.64 (0.47–0.88)	0.006
Female	67/293	54/304	0.86 (0.57–1.30)	0.482
Body mass index				
< 23	81/324	64/363	0.73 (0.50–1.05)	0.092
≥ 23	104/412	72/422	0.72 (0.51–1.01)	0.058
Smoking status				
No	109/418	74/481	0.64 (0.46–0.89)	0.008
Yes	76/318	62/304	0.84 (0.57–1.23)	0.364
Alcohol consumer				
No	103/434	85/450	0.82 (0.59–1.13)	0.220
Yes	82/302	51/335	0.59 (0.40–0.87)	0.008
Hypertension				
No	136/514	97/601	0.64 (0.47–0.85)	0.003
Yes	49/222	39/184	0.95 (0.59–1.54)	0.829
History of pancreatitis				
No	177/704	135/777	0.72 (0.56–0.93)	0.012
Yes	8/32	1/8	0.90 (0.07–10.97)	0.937
History of diabetes mellitus				
No	148/593	123/720	0.72 (0.55–0.94)	0.015
Yes	37/143	13/65	0.72 (0.35–1.48)	0.372
Family history of cancer				
No	170/704	132/775	0.72 (0.56–0.93)	0.011
Yes	15/32	4/10	0.42 (0.10–1.87)	0.258

ORs and *P* values were obtained from logistic regression models with adjustment for age, gender, body mass index, smoking status, alcohol intake, hypertension, history of pancreatitis, diabetes mellitus, and family history of cancer

### The genotype-phenotype correlation between rs1859168 and HOTTIP expression

**Fig. 1:**
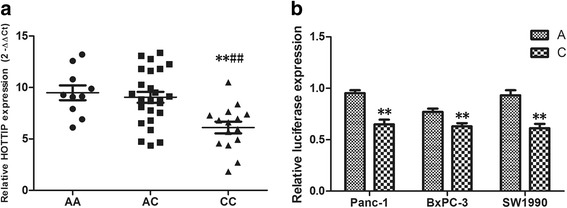
The genotype-phenotype association analysis and luciferase reporter assay. **a** The relative expression of HOTTIP with different genotypes (compared with AA genotype, ^**^ *P* < 0.001; compared with AC genotype, ^##^ *P* < 0.001). AA: *N* = 10, AC: *N* = 25, CC: *N* = 15. **b** The effect of rs1859168 on HOTTIP transcriptional activity as determined by luciferase reporter assay (compared with A allele, **P < 0.001)

In order to assess the genotype-phenotype of rs1859168 in PC, we then calculated the HOTTIP expression in PC tissue samples with different genotypes. As shown in Fig. 1a, HOTTIP expression level was significantly lower in samples with CC genotype than that in samples with AA and AC genotype (*P* < 0.001).

### The influence of rs1859168 and HOTTIP transcription activity

To further understand the mechanism of genotype-phenotype correlation, the luciferase transfection system was performed to assess the effects of rs1859168 on the promoter activity. As illustrated in Fig. 1b, the luciferase activity of the cells containing rs1859168 C allele was significantly lower than that of the cells containing rs1859168 A allele in three cell lines tested, which indicate that rs1859168 C allele may associate with a lower HOTTIP level than the rs1859168 A allele.

## Discussion

In the study, the association between important SNPs in three key lncRNA genes (HOTTIP, HOTAIR, and H19) and the risk of PC was analyzed using discovery and validation approach, and the function of HOTTIP rs1859168 A > C was also evaluated. This is the first assessment about the association between three common SNPs in critical lncRNAs (HOTTIP rs1859168 A > C, HOTAIR A > G, and H19 rs217727 C > T) and PC risk. Consistent with the results of validation set and pooled analysis, the results of discovery set showed rs1859168 carrying C allele in HOTTIP gene was significantly correlated with reduced PC risk, suggesting that the polymorphism of rs1859168 locus was significantly correlated with the incidence of PC. To explore the association between rs1859168 locus and PC, all samples in the discovery set and validation set were classified based on general characteristics. Analysis results showed a significant association under recessive model (AA/AC + CC) between rs1859168 and the risk of PC in subgroup populations with age longer than 60, males, non-smokers, drinkers, and populations with hypertension, no history of pancreatitis, no history of diabetes mellitus, and no family history of cancer. To explore the function of rs1859168, the expression of HOTTIP in PC tissues with different genotypes was measured using RT-PCR and luciferase reporter gene assay, and the results showed C allele was meaningfully correlated to the decreased HOTTIP expression. Taken together, these data suggest that the rs1859168 locus is an important factor in assessing the risk of PC and the expression of HOTTIP.

In the discovery set of this study, three locus (HOTTIP rs1859168 A > C, HOTAIR rs4759314 A > G, and H19 rs217727 C > T) on three lncRNAs referred to the initiation and progress of PC was explored. Our results indicated that neither HOTAIR rs4759314 A > G nor H19 rs217727 C > T was significantly correlated to the risk of PC. Numerous researchers have explored the relationship between these two sites and the risk of a variety of tumors. However, the results were not consistent. Our study explored the association of two loci with the risk of PC for the first time. Although they cannot be used as screening markers for PC in Chinese Han population, what surprised us was that HOTTIP rs1859168 A > C is strongly associated with the occurrence of PC.

HOTTIP, located at the 5′ end of the HOXA gene cluster, is an antisense non-coding transcript. To date, a great number of studies have reported the role of HOTTIP in pancreatic cancers. For example, a study by Cheng observed that knockdown of HOTTIP had decreased proliferation, induced apoptosis and decreased migration of PC cells [20]. Moreover, there is increasing evidence about upregulated HOTTIP in various types of cancer tissues and that was associated with cancer progression and poor prognosis for PC [34–39]. These evidences showed a tumor-promoting role of HOTTIP.

rs1859168, which is located at HOTTIP, was suggested to affect transcription factor binding sites and later the centroid secondary structure and minimum free energy, which might influence the HOTTIP expression and function [29]. In the present study, we first evaluated the association of HOTTIP rs1859168 A > C with PC risk, the study is a two-stage case-control study with large sample size provided plenty statistical power and decreased the false-positive report probability. The study showed that the C in rs1859168 had a significantly decreased risk of PC in all genetic models, suggesting this site has the potential to be a marker for PC screening. There were only two studies that have evaluated the associations of HOTTIP rs1859168 A > C with cancer risk. Gong and his colleagues performed a case-control study with a total of 498 lung cancer patients and 213 controls, and found patients carried HOTTIP rs1859168 A allele had higher lung cancer risk [29]. Similarly, our work indicated that patients carrying AA + AC have more hazards developing PC compared with individuals carrying CC.

However, a study by Richards showed that HOTTIP rs1859168 was not related to epithelial ovarian cancer risk in allele model in 1201 epithelial ovarian cancer patients and 2009 controls [40]. Extensive research has proved the expression of HOTTIP is closely related to the occurrence and development of lung cancer and pancreatic cancer, whereas no data exist concerning the relationship between the expression of HOTTIP and epithelial ovarian cancer. Thus, tumor type may be the main factor affecting the HOTTIP rs1859168 as a molecular marker for tumor screening, so as the study population. The study by Gong et al. [29] and the present work are both based on the Chinese Han population, while the research by Edward J. Richards is not, therefore, we conclude that HOTTIP rs1859168 can be used as a marker for PC in Chinese Han population, but it is not a broad-spectrum tumor marker. To further investigate the relationship between this site and the risk of pancreatic cancer, the case-control was classified based on general characteristics and analysis results showed the recessive model of rs1859168 (AA/AC + CC) was a well diagnosis biomarker of risk of PC in populations with age longer than 60, males, non-smokers, drinkers, and populations with hypertension, no history of pancreatitis, no history of diabetes mellitus, and no family history of cancer, which were not consistent with that of research in lung cancer by Gong. Therefore, attention should be paid to the population difference when using HOTTIP rs1859168 as the screening marker of PC. The work laid a good foundation for the genetic counseling of PC in future.

As the association of HOTTIP rs1859168 with the risk of PC has been demonstrated in this work, combined with the fact that HOTTIP expression was significantly related to the occurrence and development of PC, we hypothesize that HOTTIP rs1859168 polymorphism may affect the HOTTIP expression by altering its transcript activity and consequently correlate with the PC risk. Our results from the genotype-phenotype association analysis and luciferase activity confirmed this hypothesis.

There were several strengths and limitations in our present study. First, this is the first study to evaluate the association between three common SNPs in key lncRNAs genes and PC risk, and found positive association between HOTTIP rs1859168 polymorphism and PC risk. Second, the two-stage case-control study with large sample size provided plenty statistical power and decreased the false-positive report probability. Third, the functional studies of genotype-phenotype analysis and luciferase activity reporter assay mad a plausible biologic explanation of how HOTTIP rs1859168 variant affected PC risk. However, the study was hospital-based case-control study and all subjects were ethnic Han Chinese. Thus, well-designed population-based prospective studies with different ethnicities are needed to validate these results.

## Conclusions

In summary, our study provided the first evidence that the functional rs1859168 A > C polymorphism may decrease the PC risk by downregulating the HOTTIP expression levels. Extensive functional researches and additional well-designed population-based prospective studies with different ethnic groups are warranted to confirm and extend our findings.

## Abbreviations

CI: Confidence interval
lncRNAs: Long non-coding RNAs
PC: Pancreatic cancer
SNPs: Single-nucleotide polymorphisms
